# Study of the protective effect on intestinal mucosa of the hydrosoluble fiber *Plantago ovata* husk

**DOI:** 10.1186/s12906-015-0827-9

**Published:** 2015-08-29

**Authors:** Ana M. Sahagún, José Vaquera, Juan J. García, Ángela P. Calle, María-José Diez, Nélida Fernández, Juan F. Loro, Hugo O. Portilla, Matilde Sierra

**Affiliations:** Pharmacology, Department of Biomedical Sciences, Institute of Biomedicine (IBIOMED), University of Leon, Campus de Vegazana s/n, 24071 León, Spain; Department of Molecular Biology, University of Leon, Campus de Vegazana s/n, 24071 León, Spain; Pharmacology, Department of Clinical Sciences, University of Las Palmas de Gran Canaria, Campus de San Cristóbal, 35016 Las Palmas de Gran Canaria, Spain

**Keywords:** *Plantago ovata* husk, Intestinal lessions, Fiber, Anti-ulcerogenic effect, Rabbits

## Abstract

**Background:**

Several studies have indicated that dietary fiber may have a protective effect on gastrointestinal mucosa. The aim of this study was to evaluate the protective action of the soluble fiber *Plantago ovata* husk against intestinal damage.

**Methods:**

To evaluate the anti-ulcerogenic effect on duodenal mucosa of the soluble fiber *Plantago ovata* husk, low-dose acetylsalicylic acid (10 mg/kg) was given orally to animals once daily for 14 or 28 days with and without *Plantago ovata* husk (100 mg/kg). 24 h after final dosing duodenal samples were removed for anatomopathological evaluation. Villi were examined by both light and scanning electron microscopy.

**Results:**

Acetylsalicylic acid induced severe lesions in duodenal mucosa of rabbits, including erosions, epithelium disorganization, and cell vacuolization, increasing as well the amount of mononuclear and caliciform cells. Damage was much more severe in animals treated for 28 days. In groups receiving *Plantago ovata* husk, a significant attenuation of acetylsalicylic acid-induced lesions was already observed in group treated for 14 days, becoming more evident in those treated for 28 days, all of them with duodenal cytoarchitecture normal and similar to control animals.

**Conclusions:**

These findings suggest that *Plantago ovata* husk may protect intestinal mucosa probably by limiting acetylsalicylic acid penetration into epithelial cells, although further studies are needed to confirm the same effect in other experimental models of induced mucosal damage and to elucidate the mechanisms of fiber protection.

## Background

Dietary fiber can be defined as an edible component of all plants which is resistant to digestion and absorption in the human gut but available for total or partial fermentation in the large intestine [1–3]. Health benefits of high dietary fiber intake have been documented for centuries, although it is not until recent decades that fiber supplements have gained increasing attention [4–7].

According to their physico-chemical properties, dietary fibers are classified as hydrosoluble, soluble or viscous (pectin, gum, mucilage, *Plantago ovata* husk) and water-insoluble, insoluble or non-viscous (cellulose, hemicellulose, lignin) [8]. Regarding *Plantago ovata*, it is a plant which has been used traditionally in medicine because of its high content of fiber. The outest coating of *Plantago ovata* seeds (also termed as *Plantago ovata* husk or psyllium husk), obtained by milling of these seeds, is an excellent source of soluble fiber, and has become one of the most widely consumed fiber supplements, as it is well tolerated, relatively inexpensive and available in several galenic forms. Many beneficial health-related biological properties have been attributed to *Plantago ovata* husk. Among them, this fiber has been shown to prevent constipation [9, 10], diarrhea [11], Crohn’s disease [12], obesity [13], hypercholesterolemia [14–17], diabetes [17, 18] and atherosclerosis [19].

Duodenal ulcer is one of the most common gastrointestinal disorders all over the world, with an incidence of 0.04 % in the general population [20, 21]. It is developed when there is an imbalance between injurious factors (*Helicobacter pylori*, HCl, pepsins, non-steroidal anti-inflammatory drugs (NSAID), bile acids, ischemia, hypoxia, smoking or alcohol) and protective factors (bicarbonate, mucus layer, mucosal blood flow, growth factors and prostaglandins) at the luminal surface of the epithelium [22], being *Helicobacter pylori* and the widespread use of conventional NSAID such as acetylsalicylic acid the leading causes of this pathology [23, 24].

Several studies have suggested that a high fiber intake, especially soluble fibers, would have a mucosa-protective action, reducing the risk or promoting a faster healing of duodenal ulcers [25, 26]. Satoh et al. [27] have shown that diet supplementation with soluble fibers protects the small intestine against NSAID-induced damage in cats, but this study has been carried out over a short period of time (3 days). Thus, the aim of this study was to evaluate the protective action of the soluble fiber *Plantago ovata* husk against intestinal damage. To achieve this, we have used a well-known ulcerogenic agent (acetylsalicylic acid) that was orally administered for different periods of time (14 and 28 days) to rabbits.

## Methods

### Animals

Thirty healthy male New Zealand white rabbits (Granja San Bernardo, Tulebras, Navarra, Spain), weighing between 2.64 and 3.40 kg were used in this study. They were maintained in a restricted access room in the Animal Care Facility at the University of León (Spain), in metal cages which allowed the isolation of faeces in a lower container to avoid coprophagia. The environmental conditions were: humidity (55 ± 10 %), temperature (19 ± 2 °C), and a 12 h light-2 h dark cycle. Rabbits were maintained under these conditions for 7 days before the experiments. Standard laboratory chow and tap water were provided *ad libitum*. All experimental protocols were approved in advance by the Animal Care Committee at the University of León, and they were performed in accordance with the guidelines of the European and Spanish legislation.

### Treatments

The animals were randomly divided in five groups of six rabbits each. Groups I to IV were daily treated with acetylsalicylic acid (Sigma-Aldrich, St Louis, MO) by the oral route at a dose of 10 mg/kg. Groups I and II received acetylsalicylic acid for 14 days, whereas in Groups III and IV the same drug was administered for 28 days. Moreover, *Plantago ovata* husk (Plantaben®, Rottapharm SL, Barcelona, Spain) was also administered orally to Groups II and IV at a dose of 100 mg/kg, equivalent to a human dose. Finally, Group V was used as control and received only water. Animals were weighed every week in order to adjust doses of acetylsalicylic acid and fiber. Acetylsalicylic acid and *Plantago ovata* husk were administered by gastric intubation once daily every morning at the same hour. Acetylsalicylic acid was always administered dispersed in 5 ml water, followed by another 5 ml to wash the cannula. In Groups II and IV the fiber was given first, dispersed in 20 ml water, and followed by another 20 ml to remove any rest of fiber, administering then acetylsalicylic acid using the same cannula. In any of the treatments a total volume of 50 ml was used for administration and cannula cleaning.

### Histological study

Twenty-four hours after the last treatment, rabbits were sacrificed by an intravenous sodium pentobarbital overdose (200 mg/kg) (Roig Farma, Barcelona, Spain). Proximal duodenum was removed, opened with a longitudinal incision, and gently washed with saline. Samples of 2 cm each were removed from the proximal region of duodenum to perform the histological evaluation. Tissue samples were investigated by both light and scanning electron microscopy. For light microscopic studies, duodenum specimens were fixed in Bouin fluid for 48 h, dehydrated in an ascending series of ethyl alcohol, and embedded in paraffin. Approximately 5-μm-thick sections were stained with hematoxylin and eosin (H & E)/alcian blue/periodic acid Schiff for general morphology. Sections were photographed using a Nikon Eclipse microscope (Japan). For examination under scanning electron microscope, duodenum sections were fixed using 2.5 % glutaraldehyde in phosphate buffer saline (PBS) (pH 7.4). Subsequently, sample tissues were then postfixed for 2 h in 1 % osmium tetroxide in 0.05 M cacodylate buffer (pH 7.4). After fixing, tissue sections were dehydrated through a 50, 70, 90 and 100° series of acetone solutions, dried with liquid CO_2_ under pressure with critical point dryer, and covered with gold particles as preparation for examining by scanning electron microscope (JEOL JSM-6480 LV, Tokyo, Japan) in order to observe the ultrastructure of duodenal cells. A minimum of 10 images were photodocumented and analyzed from each animal at both light and scanning electron microscopic levels, and histological assessments were always made being unaware of the corresponding experimental group.

### Statistical analysis

All data are expressed as mean ± standard deviation (SD). Group comparisons were performed by using an analysis of variance (ANOVA) followed by a Student’s *t*-test. A *P* value of ≤ 0.05 was considered significant. Statistical analyses were performed with GraphPad Prism v. 4.0 (GraphPad Software, San Diego, CA).

## Results

**Fig. 1:**
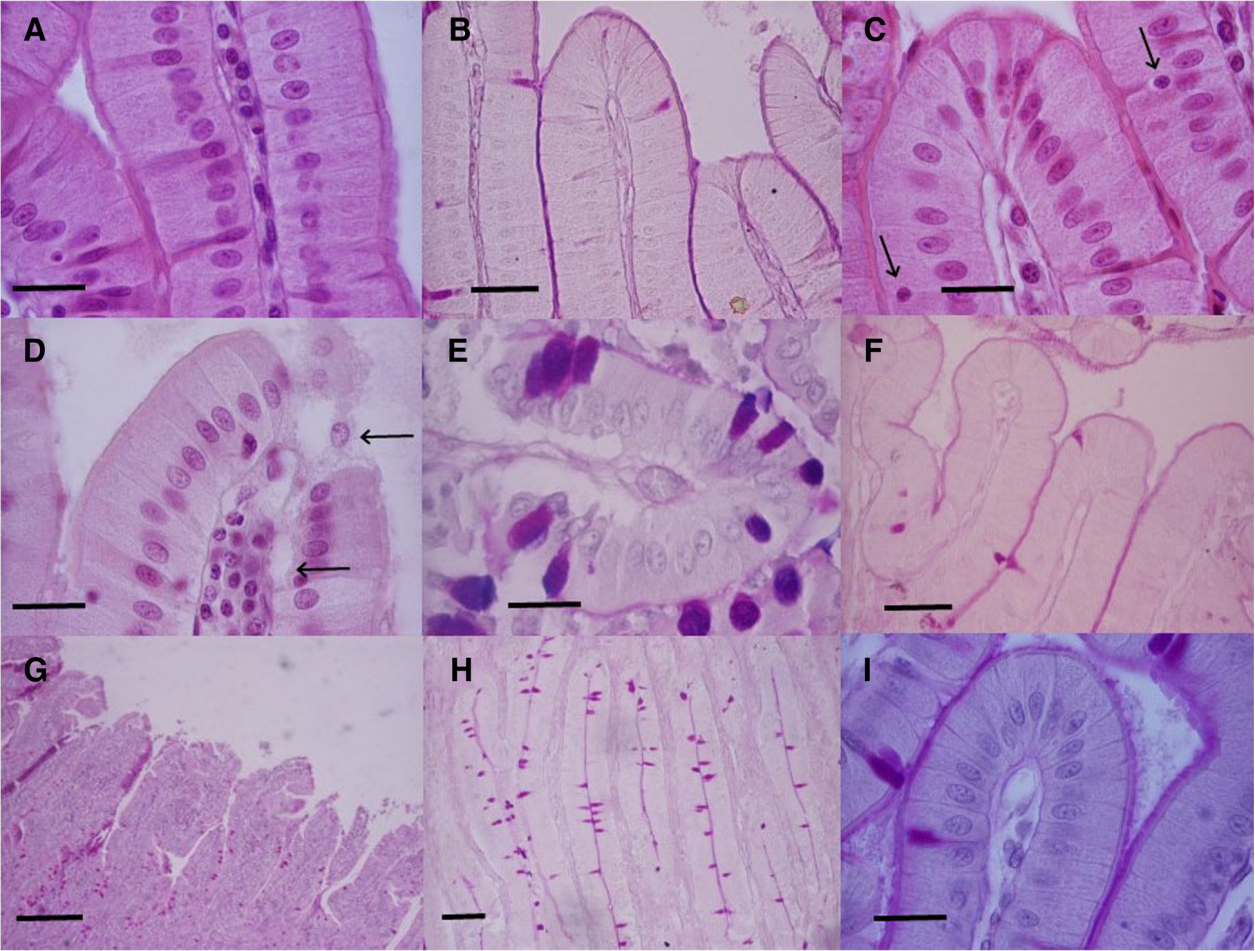
Light micrographs of rabbit duodenal mucosa showing the effect of *Plantago ovata* husk on aspirin-induced lesions. **a** and **b** Normal mucosa in control animals. **d** After 14-day aspirin treatment, epithelium disorganization and vacuolization was observed, with losses of content cell and nuclei (arrows), (**e**) also increasing the presence of caliciform cells in epithelium. In rabbits treated with fiber and aspirin for 14 days (**c** and **f**) *Plantago ovata* protected against aspirin-induced lesions, maintaining enterocyte integrity and keeping the number of caliciform cells close to control values (arrows). **g** After 28-day aspirin administration, villi are flattened and showed important erosions with 28-day treatment, and (**h**) the number of caliciform cells increased significantly, probably to augment mucin secretion. **i** Pretreatment with *Plantago ovata* husk for 28 days protected against aspirin-induced lesions, maintaining as well epithelium integrity. Hematoxyline and eosin staining: **a**, **c** and **d**, 40X, scale bar 20 μm; **g**, 4X, scale bar 100 μm. Periodic acid-Schiff (PAS) staining: **b** and **f**, 20X, scale bar 40 μm; **i**, 40X, scale bar 20 μm; **h** 10X, scale bar 50 μm. Alcian blue-PAS staining: **e** 40X, scale bar 20 μm

Control group samples showed a normal duodenal mucosa. Enterocyte layer was intact and firmly joined to underlying lamina propria, and a uniform glycocalyx was also found. Cells had a regular pattern, with spherical nuclei uniformly arranged in the basal cytoplasm (Fig. 1(a)). Compared with control animals, in rabbits treated with acetylsalicylic acid for 14 days small discontinuities were observed in glycocalix, and epithelium was disorganized and slightly vacuolized. In some samples, losses of nuclei and cell contents towards the lumen were also observed (Fig. 1(b)).

After chronic 28-day acetylsalicylic acid administration, numerous alterations in duodenal mucosa were found (Fig. 1(c)). Greater epithelium disorganization and vacuolization were noted, with more pronounced gaps. Nuclei tend to be oval-shaped and located at different heights in the medial portion instead in the basal one, due to the occurrence of blebs near the basal membrane. Moreover, villi were disorganized and flattened, with large and severe discontinuities in the apical area (Fig. 1(d)). Nevertheless, epithelium obtained from rabbits receiving acetylsalicylic acid and *Plantago ovata* husk (Figs. 1(e) and (f)) showed similar features to control samples: it was well preserved, no vacuolization was seen, and cells were firmly joined to basal lamina. Only nuclei tended to be oval-shaped and located in the basomedial region of cells.

Table 1 includes the percentage of caliciform cells, infiltrated mononuclear cells as well as the enterocytes characteristics determined in the different groups of rabbits.

**Table 1:** Percentage of caliciform cells, infiltrated mononuclear cells and enterocytes characteristics determined in the different groups of rabbits studied

	Control	AAS 14 days	AAS + fiber 14 days	AAS 28 days	AAS + fiber 28 days
	Mean ± SD	Mean ± SD	Mean ± SD	Mean ± SD	Mean ± SD
Caliciform cells (%)	13.50 ± 0.43	18.60 ± 1.40^a^	11.83 ± 0.43^a^	23.50 ± 0.87^a^	23.50 ± 0.87^a^
Infiltrated mononuclear cells (%)	1.80 ± 0.74	19.30 ± 4.50^a^	6.67 ± 0.67^a^	26.50 ± 0.76^a^	8.00 ± 0.73^a^
Enterocytes área (μm^2^)	71.61 ± 3.97	69.60 ± 1.40^a^	30.20 ± 0.65^a^	42.10 ± 1.08^a^	39.10 ± 0.95^a^
Enterocytes perimeter (μm)	36.20 ± 0.96	34.50 ± 0.33^a^	51.40 ± 1.11^a^	63.30 ± 1.53^a^	25.70 ± 0.31^a^
Enterocytes with 4 faces (%)	28	17	9	18	15
Enterocytes with 5 faces (%)	52	50	60	64	50
Enterocytes with 6 faces (%)	19	29	30	17	34

*AAS* Acetylsalicylic acid

^a^ Significant differences with control group (One way ANOVA; p ≤ 0.05)

As can be seen, in rabbits treated with acetylsalicylic acid a significant increase in the percentage of caliciform cells was observed in duodenal epithelium when compared to control group, being 18.7 % after 14-day treatment and 23.5 % following 28-day administration, while in control animals the value was 13.5 % (*P* < 0.05). These values were reduced and back to control levels in animals treated with fiber: 11.8 % and 13.25 % with 14-day or 28-day treatment, respectively (significant differences compared with acetylsalicylic acid treatments, *P* < 0.05).

Mononuclear infiltrated cells in villi also augmented with increasing times of exposure to acetylsalicylic acid, rising significantly its percentage from 1.8 % in control group to 19.3 % and 23.8 % in animals treated with this drug for 14 and 28 days, respectively (*P* < 0.05). Again, infiltrated cells diminished to 6.7 % (14 day-treatment) and 8 % (28-day treatment) in rabbits receiving *Plantago ovata* husk concomitantly. Although these values were close to those obtained in control animals, significant differences were observed when comparing to control group and acetylsalicylic acid groups (*P* < 0.05).

**Fig. 2:**
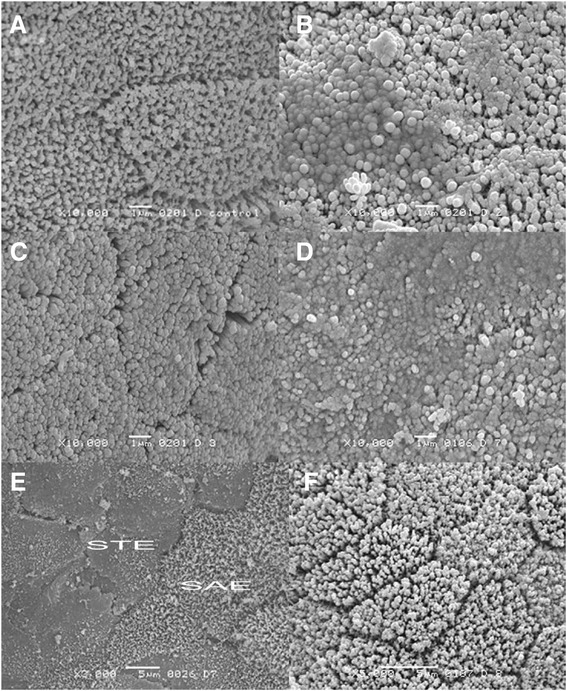
Scanning electron micrographs of rabbit duodenal mucosa, showing ultraestructural changes in enterocytes. **a** Villi of normal duodenal mucosa (scale bar = 1 μm, original magnification 10000x). **b** Villi of animals treated with acetylsalicylic acid for 14 days, with microvilli fused (scale bar = 1 μm, original magnification 10000x). **c** Villi of rabbits treated with *Plantago ovata* husk + acetylsalicylic acid for 14 days, with clearly defined limits in enterocytes (scale bar = 1 μm, original magnification 10000x). **d** Villi of animals treated with acetylsalicylic acid for 28 days, with microvilli aggregated (scale bar = 1 μm, original magnification 10000x). **e** Villi of animals treated with acetylsalicylic acid for 28 days, with slightly affected enterocytes (SAE) and strongly affected cells (STE). When several strongly affected enterocytes were adjacent, it is not possible to delimitate cells (scale bar = 5 μm, original magnification 2000x). **f** Villi of rabbits treated with *Plantago ovata* husk + acetylsalicylic acid for 28 days. Cell limits are well defined and exhibited no changes (scale bar = 5 μm, original magnification 5000x)

Finally, scanning electron microscopy revealed a normal topography of duodenal enterocytes in control group (Fig. 2(a)). In those animals that underwent treatment with acetylsalicylic acid, three types of enterocytes were observed: cells with no alterations, slightly affected enterocytes (SAE) and those strongly affected (STE). In SAE, a clear separation among cells was observed, although intercellular spaces became wider in comparison with control group, and microvilli appeared to be fused. When STE are considered, cells could be differentiated only if they were surrounded by healthy or SAE enterocytes, but if several STE were adjacent, they appeared fused among themselves. In animals treated with acetylsalicylic acid for 14 days (Fig. 2(b)), these three types of enterocytes were uniformly distributed in villi, whereas in those receiving the same treatment for 28 days (Fig. 2(d) and (e)), STE were more frequent, with duodenal microvilli almost completely fused as a continuous smooth surface. In contrast, microvilli were well defined in specimens obtained from those animals treated with *Plantago ovata* husk and acetylsalicylic acid: they were similar to those observed in the control group as well, without apparent signs of damage (Fig. 2(c) and (f)).

As we have mentioned above, Table 1 includes the characteristics obtained for enterocytes: area, perimeter and shape (number of faces). The area of the enterocytes was significantly lower in the groups of animals treated with acetylsalicylic acid and fiber for 14 days (30.2 μm^2^) or 28 days (39.1 μm^2^). Regarding the perimeter, the lowest value was found in the group of animals treated with acetylsalicylic acid and fiber for 28 days (25.7 μm). The shape of the enterocytes was evaluated taking into account the number of faces of the cell. The number of enterocytes with 5 faces was similar in all groups of animals and represented at least the 50 % of the cells. The highest percentage of cells with 4 faces was observed in the control group (28 %) and with 6 faces in the group of animals treated with acetylsalicylic acid and fiber for 28 days (34 %).

## Discussion and conclusions

Animal models of any human disease are used to mimic human conditions as much as possible in order not only to understand that disease but also to evaluate potential treatments. Among the different models of experimentally induced gastrointestinal lesions, the NSAID model is frequently used [28]. The present study was designed to address whether the hydrosoluble fiber *Plantago ovata* husk would prevent the development of duodenal lesions induced when both fiber and acetylsalicylic acid have been chronically administered.

Our study shows that severe duodenal damage may be induced by low-dose acetylsalicylic acid administration, resulting in the loss of surface epithelium and massive mononuclear infiltration. Moreover, injuries become more severe with increasing time of exposure to this drug: villi were more shortened, enterocytes were more disorganized, and structural alterations in duodenal mucosa (such as villi erosions, epithelium disorganization or blebs occurrence) were much more severe after 28-day treatment, rising significantly the amount of caliciform and infiltrated mononuclear cells as well. Severe lesions have also been reported in small intestine of rats with short-term administration of acetylsalicylic acid [29] and indomethacin [27, 30], and other authors reported an increase of mononuclear cell infiltration in gastrointestinal mucosa after having administered NSAID [31–33]. Lesions described in our study are also in accordance with the results reported by other authors in human patients, in which a higher risk of duodenal or upper gastrointestinal bleeding after chronic administration of low-dose acetylsalicylic acid was described [34, 35].

Previous studies carried out in laboratory animals and patients pointed out that several mechanisms could contribute to acetylsalicylic acid damage on the small intestine mucosa. On the one hand, the drug has a direct irritant action on the intestinal epithelium, which is considered a determinant factor in the initiation of damage. As consequence of the breach in the intestinal barrier, the release of different mediators involved in the inflammatory response would attract neutrophils into the epithelium [36, 37]. The increase in the amount of infiltrated mononuclear cells (and subsequent mucosa inflammation) in our study would confirm this hypothesis. Intestinal mucin secretion, which plays a key role in gastrointestinal protection, would also be inhibited by acetylsalicylic acid [38]. The lack of mucus on the mucosa allows luminal acid to penetrate and damage the basal lamina, retarding re-epithelization process [39]. Our results are also in accordance with this latter hypothesis, as the amount of caliciform cells is significantly augmented in animals receiving acetylsalicylic acid, probably to compensate for lower mucus secretion due to this NSAID.

Taking into account the results of this study, it is evident that long-term administration of *Plantago ovata* husk together with acetylsalicylic acid ameliorates the morphological signs of duodenal mucosal damage induced by this NSAID. This fiber tends to maintain the cytoarchitecture of the duodenal mucosa in the normal arrangement of its components, blocking thus the injurious effect of acetylsalicylic acid at this level, with well-preserved enterocytes and villi, basomedial nuclei, cytoplasm without apparent alterations, and with an amount of caliciform and mononuclear cells close to those observed in control samples. Mucosal damage was clearly minimized by *Plantago ovata* husk, and we think that this fiber would develop not only a protective action against deleterious effects of acetylsalicylic acid but also a restorative one, favoring the epithelial turnover and regeneration. In this sense, fiber was able to stimulate proliferation in the gastric glands directly [40], and fractions of *Plantago ovata* strongly stimulated the proliferation of keratinocytes [41].

We have not found any other study in which the protective effect of *Plantago ovata* husk against acute or chronic damage caused by NSAID in the small intestine has been described. In large bowel, *Plantago ovata* resulted as effective as mesalamine to maintain remission in ulcerative colitis [12] and preserved intestinal epithelium in a colitis experimental model carried out in HLA-B27 rats, with an increase in mucin secretion, a reduction of oedema and a lesser infiltration of mononuclear cells [42]. Regarding small intestine, food supplementation with soluble dietary fibers (pectin, guar gum or polydextrose) prevented the formation of duodenal ulcers induced by indomethacin when treatment was administered for 3 days to cats [27].

Several mechanisms can be involved in the protective effects of *Plantago ovata* husk observed in the intestinal epithelium. Due to the ability of this fiber to fix organic and inorganic substances, their absorption by the intestinal epithelium is delayed, reduced or even avoided, protecting this epithelium from a potential damage [43, 44]. So, fiber can retain acetylsalicylic acid, reducing the lessions observed in the epithelium. On the other hand, *Plantago ovata* husk is a highly hydrosoluble fiber, and makes markedly viscous solutions, increasing the thickness of the intestinal diffusion barrier. This effect would help to maintain the morphofunctional integrity of intestinal epithelium by preventing or slowing acetylsalicylic acid penetration and trapping in the intestinal mucosa and, consequently, the topical action and the extensive lesions induced by this drug at this level. Moreover, viscosity is also associated with prolonged gastric emptying [45], increasing the retention of acetylsalicylic acid in stomach and, consequently, delaying the access of this drug to intestinal mucosa.

Dietary fibers may also protect duodenal mucosa by decreasing gastric acid secretion [26, 46], diminishing the load of harmful acid that could have access to duodenal mucosa. Additional mechanisms may include an anti-inflammatory action, reducing proinflammatory biomarkers such as C-reactive protein, IL-6, IL-12 or tumor necrosis factor-α [47, 48].

Taking into account the results obtained in this study, we can conclude that *Plantago ovata* husk, administered at the same time as acetylsalicylic acid, avoids the gastrointestinal lesions caused by this drug. This fact can be due to the solubility and viscosity of the fiber, or due to a reduction in the absorption of acetylsalicylic acid, although further studies are needed to confirm the same effect in other experimental models of induced mucosal damage and to elucidate the mechanisms of fiber protection.
